# Spectral data of specular reflectance, narrow-angle transmittance and angle-resolved surface scattering of materials for solar concentrators

**DOI:** 10.1016/j.dib.2015.11.059

**Published:** 2015-12-11

**Authors:** Philipp Good, Thomas Cooper, Marco Querci, Nicolay Wiik, Gianluca Ambrosetti, Aldo Steinfeld

**Affiliations:** a Department of Mechanical and Process Engineering, ETH Zurich, 8092 Zurich, Switzerland; b Airlight Energy Manufacturing SA, 6710 Biasca, Switzerland

**Keywords:** Solar materials, Reflectance, Transmittance, Specular, Angular scattering, Solar concentrators

## Abstract

The spectral specular reflectance of conventional and novel reflective materials for solar concentrators is measured with an acceptance angle of 17.5 mrad over the wavelength range 300−2500 nm at incidence angles 15–60° using a spectroscopic goniometry system. The same experimental setup is used to determine the spectral narrow-angle transmittance of semi-transparent materials for solar collector covers at incidence angles 0–60°. In addition, the angle-resolved surface scattering of reflective materials is recorded by an area-scan CCD detector over the spectral range 350–1050 nm. A comprehensive summary, discussion, and interpretation of the results are included in the associated research article “Spectral reflectance, transmittance, and angular scattering of materials for solar concentrators” in Solar Energy Materials and Solar Cells.

**Specifications Table**

**Table:** 

Subject area	Optics

More specific subject area	Spectroscopy
Type of data	Table
How data was acquired	Spectroscopic goniometry system
Data format	Raw, analyzed
Experimental factors	none
Experimental features	Specular reflectance/narrow-angle transmittance at a detector acceptance half-angle of 17.5 mrad, wavelengths 300–2500 nm, and incidence angles 15°/0–60°. Angle-resolved surface scattering at wavelengths 350–1050 nm.
Data source location	Zurich, Switzerland
Data accessibility	With this article

**Value of the data**

• These data are a complete set of optical properties of representative solar concentrator materials and can serve as a benchmark for other researchers in the field of solar energy for the accurate simulation of solar concentrators.
• The spectral data is valuable for the optimization of solar concentrating systems such as improving selective and (anti-)reflective coatings and solar cell tuning for concentrated photovoltaics.
• The angle-resolved surface scattering data is useful for the accurate simulation of the solar flux distribution on the receiver and the optical design of solar collectors with small acceptance angles such as far-distant heliostats.

## 1 Data

Spectral data of specular reflectance of solar reflector materials and narrow-angle transmittance of semi-transparent materials for solar collector covers are experimentally measured at an acceptance half-angle of 17.5 mrad, wavelengths 300–2500 nm, and incidence angles 0–60°. The angle-resolved surface scattering of reflective materials is characterized by the parameters of a superposition of two Gaussian distributions over the spectral range 350–1050 nm and incidence angles 15–60°.

## 2 Experimental design, materials and methods

### 2.1 Materials

Three types of specular reflective materials (back-silvered glasses, metallized polymer films and metallized aluminum sheets) and two types of semi-transparent materials (glasses and polymeric films) are characterized. An overview of the materials is given in Table 1. A comprehensive description of materials is included in Chapter 2 of the associated research article [1].

### 2.2 Experimental design

**Fig. 1:**
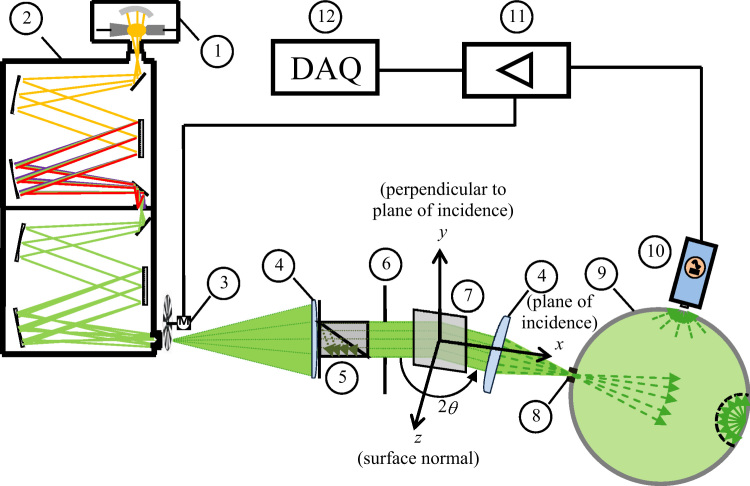
: The spectroscopic goniometry system comprised of: (1) Xe-arc lamp, (2) double monochromator, (3) chopper, (4) imaging lens pair, (5) polarizer, (6) iris, (7) sample, (8) mechanical slit, (9) integrating sphere, (10) photodetector, (11) lock-in amplifier, and (12) data acquisition system. The *x–y–z* coordinate system is centered at the pivot point and *x–z* defines the plane of incidence [1].

Spectral measurements are performed using a spectroscopic goniometry system [15]. The experimental design for measuring specular reflectance and narrow-angle transmittance is shown schematically in Fig. 1. Following the light path, the setup comprises a xenon-arc light source (1), aspherical Czerny–Turner type double monochromator (2), mechanical beam chopper (3), collimating MgF_2_ lens (4), calcite Glan–Thompson polarizer (5), iris (aperture stop) (6), sample (7), focusing MgF_2_ lens (4), adjustable mechanical slit (field stop) (8), integrating sphere (9), thermoelectrically cooled photodiode detector (Si: 300−1000 nm, PbS: 1000−2800 nm) (10), lock-in amplifier (11), and computer based data acquisition system (12). The source divergence and detector acceptance half-angles are *θ*_src,*x*_=6.0 mrad and *θ*_acc,*x*_=17.5 mrad in the plane of incidence and *θ*_src,*y*_=8.0 mrad and *θ*_acc,*y*_=50 mrad in the plane perpendicular to the plane of incidence, respectively. For the characterization of angle-resolved surface scattering, the source divergence half-angle in the plane of incidence is reduced to *θ*_src,*x*_=0.31 mrad and the detector assembly (adjustable slit+integrating sphere+photodetector) is replaced by an area scan CCD camera with field of view *θ*_acc,*x*_=13.0 mrad by *θ*_acc,*y*_=9.66 mrad and angular resolution 0.033 mrad. A detailed description of the experimental setups used for specular reflectance/narrow-angle transmittance and angle-resolved surface scattering is provided in Sections 3.1 and 4.1 of the associated research article, respectively [1].

### 2.3 Methods

For each spectral data point, three sequential measurements are performed at the same wavelength: (1) a reference measurement with the sample moved out of the beam and the detector arm positioned at 180°; (2) a sample measurement with the sample placed in the source beam at an incidence angle *θ* and the detector arm rotated to the corresponding angular position (2*θ* for reflection; 180° for transmission); and (3) a second reference measurement.

#### 2.3.1 Specular reflectance and narrow-angle transmittance

The spectral specular reflectance and narrow-angle transmittance are calculated as the ratio of the voltage from the sample measurement to the average voltage from the reference measurements. For polarization-dependent optical properties the above procedure is performed twice, with the electric field of incident light oscillating once in the plane parallel to the plane of incidence (parallel or p-polarized), and once in the plane perpendicular to the plane of incidence (perpendicularly or s-polarized), and the optical property for unpolarized sunlight is calculated as the average of p- and s-polarized light [16]. Type A measurement uncertainties of spectral specular reflectance and narrow-angle transmittance are calculated from the estimated variance of the recorded voltage signals using the Gaussian error propagation formula. The maximum type A uncertainty occurring at 300 nm and the root-mean-square uncertainty over the measured spectral range are below 0.02 and 0.003, respectively. The type B uncertainty is estimated as 0.004 from spectral transmittance and reflectance measurements of an N-BK7HT glass sample with known optical properties [17]. Accordingly, the combined measurement uncertainty calculated with a confidence factor 2 (95% confidence) is usually within 0.01 for spectral specular reflectance and narrow-angle transmittance. The spectra and type A uncertainties of specular reflectance and narrow-angle transmittance are given in the data Tables A.1 and A.2, respectively.

#### 2.3.2 Angle-resolved surface scattering

The angular scattering is quantified by the parameters of statistical surface scattering models [18]. For the scattering function of silvered polymer films and silvered aluminum sheets with the manufacturing marks perpendicular to the plane of incidence, a superposition of two Gaussian distributions is used,

(1) $$f\left(\theta_{s,x}\right)=\frac{F_{1}}{\sqrt{2\pi \sigma_{1}^{2}}}\exp\left(-\frac{\theta_{s,x}^{2}}{2\sigma_{1}^{2}}\right)+\frac{1-F_{1}}{\sqrt{2\pi \sigma_{2}^{2}}}\exp\left(-\frac{\theta_{s,x}^{2}}{2\sigma_{2}^{2}}\right).$$

where *θ*_s,*x*_ is the angular deviation of the reflected ray from the perfectly specular direction, *σ*_1_ and *σ*_2_ the standard deviations of the first and second distributions, and *F*_1_ the fraction of rays following the first distribution. The parameters of *f* are identified by matching the convolution of the scattering function and the measured reference beam shape to the scattered beam shape of the sample measurement using the least-square technique. The method is described in more detail in Section 4.2 of the associated research article [1]. The identified parameters at different wavelengths and incidence angles are included in Table B1.

## Figures and Tables

**Table 1:** Reflective and semi-transparent solar materials considered in this study [1].

Sample name	Product description	Thickness	Intended environment	Performance reported by manufacturer	
*Back-silvered glass*				*R*_direct,solar_[2]	
AgGlass4 mm	Flat glass mirror (2014)	4 mm	Outdoor	≥0.945 [3]	
AgGlass2 mm	Flat glass mirror (2013)	2 mm	Outdoor	≥0.945 [3]	
AgGlass1 mm	Flat glass mirror (2008)	1 mm	Outdoor	≥0.945 [3]	

*Metallized polymer films*				*R*_h,solar_	*R*_specular, 660 nm,15°,25 mrad_
AgFilm#1	Silvered acrylic film	117 μm	Outdoor	0.94	> 0.95 [4]
AgFilm#2	Silvered polymer film	100 μm	Outdoor	0.93	0.94 [5]
AlFilm	Aluminized boPET	23 μm	Indoor	[6]	

*Metallized aluminum sheets*				*R*_h,solar/light_	*R*_specular, 60° ISO 7668_
AgSheet#1	Silvered aluminum sheet	0.5 mm	Indoor	≥0.95	≥0.92 [7]
AgSheet#2	Silvered aluminum sheet	0.4 mm	Indoor (lighting)	≥0.98	≥0.93 [8]
AgSheet#3	Silvered aluminum sheet	0.3 mm	Indoor (lighting)	≥0.98	≥0.93 [9]
AlSheet	Aluminized aluminum sheet	0.4 mm	Outdoor	≥0.89	≥0.88 [10]

Transparent polymer films					
ETFE100 μm	ETFE film	100 μm	Outdoor	[11]	
FEP100 μm	FEP film	100 μm	Outdoor	[12]	

*Transparent glass*				*T*_normal,solar_	
Borosilicate3.3 mm	borosilicate substrate	3.3 mm	Outdoor	0.92 [13]	
BorosilicateAR3.3 mm	AR-coated borosilicate	3.3 mm	Outdoor	0.97 [14]	

## Footnotes

Appendix A Supplementary data associated with this article can be found in the online version at doi:10.1016/j.dib.2015.11.059.
